# The Art Amateur for September

**Published:** 1894-10

**Authors:** 


THE ART AMATEUR FOR SEPTEMBER. Published by Montague Marks, No. 23 Union Square, New York.

Reviews have already been given of former numbers of this interesting monthly devoted to art in the household. The frontispiece is a double portrait study in crayon, by Paul Merwart. The article entitled, "Bruce Crane and His Work," is well illustrated, and the portrait of Paul Renourd will be of interest to artists. The supplement contains five designs. "Snipe" is the seventh of a set of twelve game plates. Chair back for wood carving, decoration of a tea set in Dresden style, embroidered border for a portière, and scroll letters for embroidery. The drawing study is called "The Parting Shot," after the painting by Frederick Morgan. The color plates are an "Indian Summer," by Bruce Crane, and "Carnations," by Paul de Longpre.

